# Assessing the performance of monocyte to high-density lipoprotein ratio for predicting ischemic stroke: insights from a population-based Chinese cohort

**DOI:** 10.1186/s12944-019-1076-6

**Published:** 2019-05-30

**Authors:** Hao-Yu Wang, Wen-Rui Shi, Xin Yi, Ya-Ping Zhou, Zhi-Qin Wang, Ying-Xian Sun

**Affiliations:** 1 Department of Cardiology, The First Hospital of China Medical University, 155 Nanjing North Street, Heping District, Shenyang, 110001 China; 2 Department of Cardiology, State Key Laboratory of Cardiovascular Disease, Fuwai Hospital, National Center for Cardiovascular Diseases, Chinese Academy of Medical Sciences and Peking Union Medical College, Beijing, China; 3 Department of Cardiovascular Medicine, Beijing Huimin Hospital, Beijing, 100054 China; 4 Department of Neurology, The First Hospital of China Medical University, 155 Nanjing North Street, Heping District, Shenyang, 110001 China; 5 School of Clinical Medicine, China Medical University, Shenyang, 110122 China

**Keywords:** Monocyte to high-density lipoprotein ratio, Ischemic stroke, Atherosclerosis, Dyslipidemia, Inflammation, Monocyte, Epidemiology

## Abstract

**Background:**

Monocyte to high-density lipoprotein cholesterol ratio (MHR) is a recently emerged measure of inflammation and oxidative stress and has been used to predict multiple cardiovascular abnormalities, but data relative to ischemic stroke are lacking. The goal of this study was to estimate the associations of MHR and prevalent ischemic stroke among a large cohort of general Chinese population.

**Method:**

The study analyzed 8148 individuals (mean age: 54.1 years; 45.7% males) enrolled in a cross-sectional population-based Northeast China Rural Cardiovascular Health Study (NCRCHS). We identified 194 patients admitted from January and August 2013 with ischemic stroke.

**Results:**

After adjustment for age, sex, and potential confounders, each standard deviation (SD) increment of MHR was predictive to a greater odd of ischemic stroke (odds ratio, 1.276; 95% confidence interval [CI], 1.082–1.504), with subjects in the highest quartile of MHR levels having a 1.6-fold higher risk of prevalent ischemic stroke (95% CI, 1.045–2.524) as compared with those in the lowest quartile. Moreover, smoothing curve showed a linear positive pattern of this association. The area under the curve (AUC) significantly increased (*P* = 0.042) to 0.808 (95% CI, 0.779–0.837) when the combined MHR was added to the baseline logistic regression model with ischemic stroke risk factors. Also, MHR (0.004) significantly improved integrated discrimination improvement when added to the baseline model.

**Conclusions:**

The present study demonstrated for the first time a linear relation between MHR levels and the odds of ischemic stroke in a large community-based population. The MHR, a marker of high atherosclerotic burden, demonstrated incremental predictive value over traditional clinical risk factors, thus providing clinical utility in risk stratification in subjects presenting with ischemic stroke. These findings had implications for strategies aimed at lowering MHR to prevent adverse cardiovascular and cerebrovascular outcomes.

## Introduction

Over the past decades, stroke has emerged as a major burden of healthcare system [1]. In 2013, stroke affected 1596 per 100,000 people and attributed to 114.8 new deaths per 100,000 people in China [2]. Moreover, the burden of stroke in China appears to be increasing particularly in rural areas, of which ischemic stroke constituted 69.6% [2, 3]. Indeed, ischemic stroke has become a predominant cause of death and disability in the low income population [4]. There is thus a clear need to identify ischemic stroke at an early stage, and as a result, primary prevention forms the cornerstone of management.

Atherosclerosis, especially intracranial atherosclerosis, is an intrinsic abnormality in the development of ischemic stroke [5–7]. Pathophysiological studies have identified intracranial plaques can cause artery-to-artery emboli, in-situ thromboembolism, hemodynamic impairment and local branch occlusion, which play critical roles in the onset of ischemic stroke [8, 9]. Additionally, clinical studies revealed the high prevalence of intracranial atherosclerosis among ischemic stroke patients in different races through advanced imaging techniques or autopsy [10–13]. However, advanced imaging techniques are expensive, time-consuming and unavailable in resource-poor settings, and therefore, a novel marker that can be used both clinically and in research studies as intermediate or surrogate outcomes to help early identification and prevention of ischemic stroke is needed.

Inflammation and lipid abnormalities have been proposed as the main constituents of the pathophysiology of atherosclerosis development and progression [14, 15]. Monocytes, hallmarks of chronic inflammation, interact primarily with platelets and endothelial cells resulting in aggravation of inflammatory, pro-thrombotic pathways and are known to play an active role in the formation, progression and rupture of an atherosclerotic plaque at the vascular level [16, 17]. Conversely, the high-density lipoprotein-cholesterol (HDL-C) protect endothelial cells from inflammation and oxidative stress through controlling monocyte activation and proliferation of monocyte progenitor cells, as well as suppressing the migration of macrophages and oxidation of low-density lipoprotein (LDL) molecules [18, 19]. In view of the pathogenesis of atherosclerosis, HDL-C level is a lipid parameter that reduces in the presence of endothelial dysfunction and atherosclerosis, and monocyte is a hematological index that increase during inflammation. In this regard, theoretically, HDL-C levels decline while monocyte levels increase in cases of atherosclerosis, thus, the MHR value is expected to increase.

From these observations, a model constructed using these two indices, that is, the emerging monocyte-to-HDL-C ratio (MHR), has been put forward to evaluate the prognosis of cardiovascular events by assessing atherosclerosis [20]. Later studies also revealed its utility in the prediction of hallmarks of cardiovascular outcomes, such as metabolic syndrome, coronary artery disease, and atrial fibrillation recurrence [21–25]. However, no study to date has evaluate the association between MHR and ischemic stroke. Therefore, this study aimed to investigate the impact of MHR on ischemic stroke and explore the value of MHR to stratify the risk of ischemic stroke based on 8148 participants of the general population from China.

## Materials and methods

### Study population

**Fig. 1:**
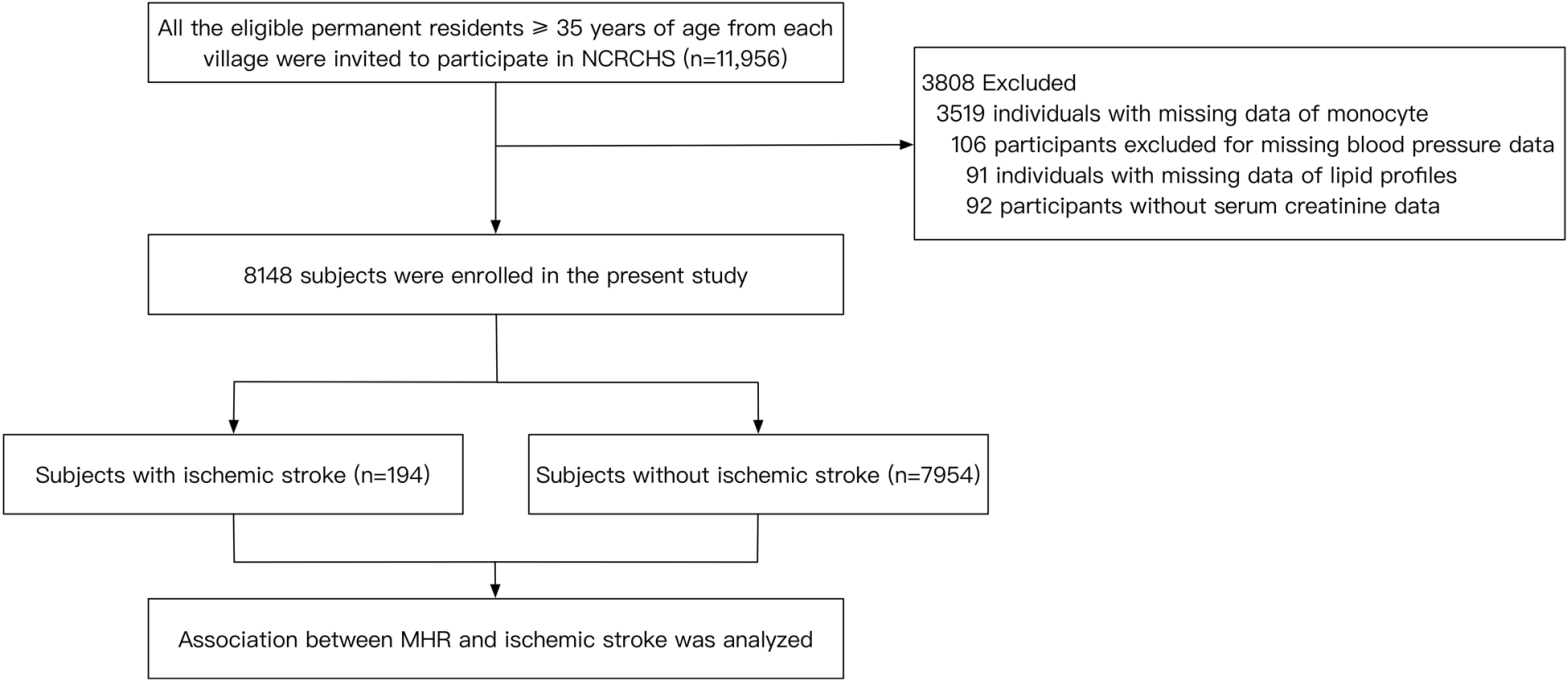
Flowchart describing the selection process and derivation of the study population

The present study originated from a large population-based cross-sectional epidemiological investigation named Northeast China Rural Cardiovascular Health Study (NCRCHS). After excluding people with pregnancy, malignant tumor and mental disease, the survey recruited a total of 11,956 participants (age ≥ 35 years) between January 2013 and August 2013 at 26 rural villages across Liaoning province in China. Details regarding the design and rationale of NCRCHS has been fully displayed elsewhere [26–28]. In the present study, 3808 participants were further excluded due to their missing values of covariates. Finally, 8148 subjects (mean age: 54.1 years, male: 45.7%) were carried into statistical analyses (Fig. 1). Our study protocol was approved by the Ethic Committee of China Medical University (Shenyang, China) and the whole data and procedures conformed to the principle of ethical standards. Every enrolled participant provided a written informed consent.

### Data collection and measurements

Prior studies have delineated data collection and measurements in detail [26–28]. In brief, after cardiologists and nursed completed a training course and passed an exam, they were certificated for conducting questionnaires which collected data about demographic information, health-related behavior, anthropometric parameters and the use of lipid-lowering drug (at least one type versus no). Subjects were asked to indicate their average use of food items during the previous year for assessment of the dietary patterns. We used a food frequency questionnaire to collect baseline information on dietary habits in terms of the average consumption of several food items per week. The reported consumption was quantified approximately according to the grams per week. Vegetable consumption was rated on the following scale: scored 0, 1, 2, and 3 for ≥2000 g, 1000-2000 g, <1000 g, and rare level, respectively. Meat consumption, including red meat, fish and poultry, was classified into 4 categories as follows: rarely = 0 g/wk., <250 g/wk. = 1, 250–500 g/wk. = 2 and ≥ 500 g/wk. = 3. A diet score was generated by adding the vegetable consumption score (from 0 to 3) plus meat consumption score (from 0 to 3) on a scale of 0–6 for each participant, with higher scores indicating greater Westernized diet adherence. Meanwhile, a central steering committee with a subcommittee was employed to fulfill the quality control process of these collected data.

Study participants waited for at least 5 min in a relaxed and sitting position. Then blood pressure (BP) was measured by a standardized automatic electronic sphygmomanometer (HEM-907; Omron, Kyoto, Japan) using an appropriately sized cuff with the arm supported at the level of the heart. The mean readings of three replicate measurements were recorded for the present analysis.

Anthropometric indexes were collected when subjects only wore light clothing without shoes. The weight of subjects was quantified to the nearest 0.1 kg by a calibrated digital scale. After subjects held in a standing position, their height was recorded by a portable stadiometer with a scale of 0.1 cm. An elastic measuring tape was utilized to obtain the waist circumference (WC) horizontally at 1 cm above the umbilicus. All of the above measurements were conducted twice and their mean values were included into the analyses. The subjects were reminded of keeping an overnight fasting with 12 h before the investigation. Details about the process of blood sample transportation, storage and laboratory tests were extensively described in our previous studies [26–28].

### Definitions

Body mass index (BMI) was calculated as mean weight divided by mean height squared (kg/m^2^). MHR was defined as blood monocyte count ratio to high-density lipoprotein cholesterol concentration [20]. Hypertension was recognized as systolic blood pressure (SBP) ≥ 140 mmHg and / or diastolic blood pressure (DBP) ≥ 90 mmHg, subjects with self-reported current antihypertensive medication or previous diagnosed hypertension were also considered as hypertensive patients [29]. Diagnosis of diabetes based on fasting plasma glucose (FPG) ≥ 7.0 mmol/L and/or self-reported previous diagnosed diabetes, subjects receiving plasma glucose lowering therapy at baseline were also regarded as diabetic patients [30].

Based on strict neurological examination, computed tomography or magnetic resonance imaging, first-ever ischemic stroke was defined as stroke event with a diagnosis of thrombosis or embolism. Documentation of ischemic stroke reviewed by two independent neurologists was confirmed on the basis of the information on diagnostic tests or hospital records.

### Statistical analyses

Continuous variables were expressed as mean values ± standard deviation (SD) or median (interquartile range) according to their distributions. Category variables were presented as frequencies (percentages). Before inferential analyses, MHR values were log transformed due to highly skewed distributions. Comparisons of continuous variables were conducted by Student’s t test or Mann-Whitney test. Chi-square test was occupied to compare categorical variables between groups. Additionally, to compare ordinal categorical variables (education level, family annual income, physical activity) between groups, rank-sum test was employed to utilize the ordinal information. Adjusted odds ratios (ORs) and 95% confidence intervals (CIs) for the independent association between MHR and the prevalent ischemic stroke were determined using a multivariate logistic regression model adjusted for potential confounding covariates. The confounding variables used for adjustment included age, sex, race, education, family annual income, physical activity status, current smoking and drinking conditions, diet score, serum creatinine (Scr), SBP, FPG, BMI, and lipid-lowering drug. Analyses were conducted with MHR as continuous variables (per SD increase) and then categorized as quartiles. The results were displayed as odds ratios (ORs) and 95% confidence intervals (95% CI). A generalized additive model (GAM) with a spline smoothing function was applied to examine the relationship between MHR and the risk of ischemic stroke. Subgroup analysis examined the relationship between MHR and the risk of ischemic stroke according to age, sex, SBP (< 140 and ≥ 140 mmHg), FPG (< 7.0 and ≥ 7.0 mmol/L), and BMI (< 28 and ≥ 28 kg/m^2^). Test for interaction in the logistic-regression model was used to compare odd ratios (ORs) between the analyzed subgroups. The incremental predictive value of MHR in addition to clinical risk factors, was assessed using the Harrell’s C index, net reclassification improvement (NRI), and the integrated discrimination improvement index (IDI). Clinical risk factors included age, sex, current smoking, current drinking, Scr, SBP, FPG and BMI. All of the statistical analyses were performed by SPSS 25.0 software (IBP corp), statistical software packages R (http://www.r-project.org, The R Foundation) and EmpowerStats (http://www.empowerstats.com, X&Y Solutions, Inc., Boston, MA). A two-tailed *P* value less than 0.05 was recognized as significant.

## Results

Table 1 summarizes the data of 8148 eligible subjects (males: 45.7%). The prevalence of ischemic stroke was 2.38%. As for the demographic data, participants with ischemic stroke were older and had lower education, family annual income and physical activity status than their healthy counterparts. Additionally, ischemic stroke subjects were less likely to be a current smoker or drinker. With regard to the anthropometric characteristics, ischemic stroke group had a significant lower level of height, with the exception of weight and BMI, which were unaltered. Concordantly, adults with ischemic stroke exhibited higher levels of SBP and DBP together with increased SCr and FPG. As expected, participants with ischemic stroke constituted a higher percentage of hypertension, diabetes, and lipid-lowering drug relative to the non-ischemic stroke ones. Regarding lipid profiles, higher total cholesterol, triglyceride and LDL-C were remarkably augmented in ischemic stroke patients compared with their counterparts, while HDL-C concentrations were greatly reduced in ischemic stroke subjects (all *P* < 0.05). Within our expectation, patients with ischemic stroke experienced pronounced increment of white blood cell count, neutrophil and monocyte counts. Lastly, MHR levels were statistically greater in ischemic stroke subjects than non-ischemic stroke group.

**Table 1:** Characteristics of subjects stratified by ischemic stroke

Variables	Ischemic stroke (*N* = 194)	Non-ischemic stroke (*N* = 7954)	*P* value ^a^
Age (years)	64.48 ± 8.87	53.87 ± 10.39	<0.001
Male (%)	86 (44.3)	3641 (45.8)	0.690
Race (Han) (%)	188 (96.9)	7740 (97.3)	0.773
Education level (%)			<0.001
Primary school or below	142 (73.2)	4171 (52.4)	
Middle school	40 (20.6)	3091 (38.9)	
High school or above	12 (6.2)	692 (8.7)	
Income (CNY) (%)			<0.001
≤5000	56 (28.9)	795 (10.0)	
5000–20,000	100 (51.5)	4183 (52.6)	
>20,000	38 (19.6)	2976 (37.4)	
Physical activity (%)			<0.001
Low	142 (73.2)	3038 (38.2)	
Middle	24 (12.4)	1522 (19.1)	
High	28 (14.4)	3394 (42.7)	
Current smoking (%)	53 (27.3)	2712 (34.1)	0.049
Current drinking (%)	21 (10.8)	1712 (21.5)	<0.001
Diet score	1.72 ± 1.15	2.28 ± 1.14	<0.001
Height (cm)	158.46 ± 8.12	160.72 ± 8.14	<0.001
Weight (kg)	62.53 ± 9.54	63.53 ± 11.31	0.153
BMI (kg/m^2^)	24.91 ± 3.41	24.53 ± 3.61	0.151
SBP (mmHg)	154.16 ± 25.31	138.29 ± 21.71	<0.001
DBP (mmHg)	85.85 ± 11.88	81.76 ± 11.60	<0.001
Scr (μmol/L)	78.60 (70.48–88.63)	73.70 (66.60–82.10)	<0.001
FPG (mmol/L)	5.84 (5.38–6.59)	5.60 (5.22–6.09)	<0.001
TC (mmol/L)	5.48 ± 1.06	5.31 ± 1.11	0.038
TG (mmol/L)	1.81 (1.17–2.69)	1.29 (0.91–1.95)	<0.001
HDL-C (mmol/L)	1.24 ± 0.28	1.34 ± 0.32	<0.001
LDL-C (mmol/L)	3.01 ± 0.80	2.89 ± 0.80	0.033
WBC count (10^9^/L)	6.30 (5.30–7.60)	6.00 (4.90–7.10)	<0.001
Neutrophil count (10^9^/L)	3.80 (3.00–4.64)	3.40 (2.70–4.30)	<0.001
Lymphocyte count (10^9^/L)	1.90 (1.60–2.40)	1.90 (1.60–2.40)	0.593
Monocyte count (10^9^/L)	0.50 (0.35–0.68)	0.41 (0.30–0.60)	0.004
Hypertension (%)	167 (86.1)	3642 (45.8)	<0.001
Diabetes (%)	46 (23.7)	828 (10.4)	<0.001
Lipid-lowering drug (%)	30 (15.5%)	242 (3.0%)	<0.001
MHR	0.39 (0.27–0.55)	0.34 (0.23–0.48)	<0.001

Data are expressed as mean ± standard deviation (SD) or median (interquartile range) and numbers (percentage) as appropriate

Abbreviations: *CNY* Chinese currency (1CNY = 0.15 USD), *BMI* body mass index, *SBP* systolic blood pressure, *DBP* diastolic blood pressure, *Scr* serum creatinine, *FPG* fasting plasma glucose, *TC* total cholesterol; *TG* triglyceride, *HDL-C* high-density lipoprotein cholesterol, *LDL-C* low-density lipoprotein cholesterol, *WBC* white blood cell, *MHR* monocyte count to high-density lipoprotein ratio

^a^Comparisons of category variables between groups were tested by chi-square test or rank-sum test (ordinal category variables) and comparisons for continuous variables between groups were tested by Student’s t or Mann-Whitney test

**Fig. 2:**
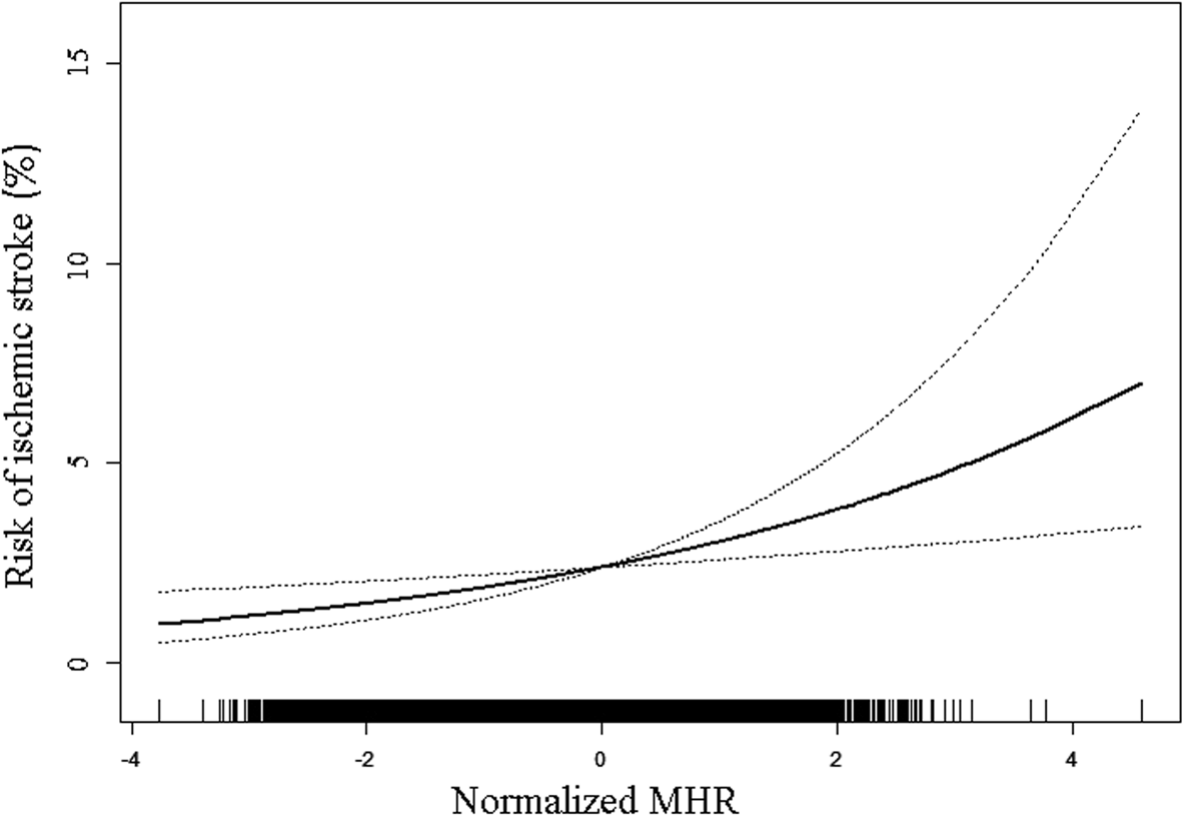
Smooth curve fitting was performed using generalized additive model to explore the association between MHR and the risk of ischemic stroke after adjusting for age, sex, race, education level, family annual income level, physical activity, current smoking, current drinking, diet score, serum creatinine, systolic blood pressure, fasting plasma glucose, body mass index, and lipid-lowering drug. In this figure, the solid line indicates the estimated risk of ischemic stroke while the dotted lines serve as pointwise 95% confidence intervals

Multivariate logistic regression model demonstrated the association between MHR and ischemic stroke (Table 2). After adjusting for age, sex, race, education, family annual income and physical activity status, current smoking and drinking conditions, and diet score, each SD increment of MHR could cast additional 37.5% risk of ischemic stroke on subjects. Additional adjustment of Scr, SBP, FPG, BMI, and lipid-lowering drug mildly modified this association, but the full model still had a OR of 1.276 (95% CI: 1.082, 1.504). After dividing MHR into quartiles, we observed a 62.4% increase of the risk for ischemic stroke when comparing top quartile with bottom category in the fully adjusted model. Furthermore, the risk of ischemic stroke showed an increasing trend across the quartiles (P for trend = 0.024). Additionally, in order to explore the dose-response relationship between MHR and the risk of ischemic stroke, we employed smooth curve fitting with full adjustment of all covariates (Fig. 2). The resultant curve displayed a linear correlation between normalized MHR and ischemic stroke risk. This result was consistent with the increasing trend across the quartiles in the logistic model.

**Table 2:** Evaluation of the impact of MHR on ischemic stroke by multivariate logistic regression models

Variables	Odds Ratio (95% CI)					
	Crude	*P* value	Model 1	*P* value	Model 2	*P* value
MHR (Per 1 SD increase)	1.426 (1.221, 1.666)	<0.001	1.375 (1.170, 1.615)	<0.001	1.276 (1.082, 1.504)	0.004
Quartiles of MHR						
Quartile 1	1.000 (reference)		1.000 (reference)		1.000 (reference)	
Quartile 2	1.314 (0.831, 2.076)	0.243	1.206 (0.756, 1.925)	0.432	1.155 (0.720, 1.851)	0.550
Quartile 3	1.434 (0.915, 2.248)	0.116	1.393 (0.880, 2.207)	0.157	1.257 (0.788, 2.005)	0.338
Quartile 4	2.186 (1.440, 3.319)	<0.001	1.924 (1.252, 2.956)	0.003	1.624 (1.045, 2.524)	0.031
P for trend		<0.001		0.001		0.024

Abbreviations: *MHR* monocyte count to high-density lipoprotein cholesterol ratio, *OR* odds ratio; 95% CI: 95% confidence interval, *SD* standard deviation. Other abbreviations as in Table 1

Crude: no adjustment; Model 1: adjusted for age, sex, race, education level, family annual income level, physical activity, current smoking, current drinking, and diet score; Model 2: adjusted for all the factors in model 1 and serum creatinine, systolic blood pressure, fasting plasma glucose, body mass index, and lipid-lowering drug

Quartile 1: MHR < 0.229; Quartile 2: 0.229 ≤ MHR < 0.341; Quartile 3: 0.341 ≤ MHR < 0.485; Quartile 4: MHR ≥ 0.485

**Fig. 3:**
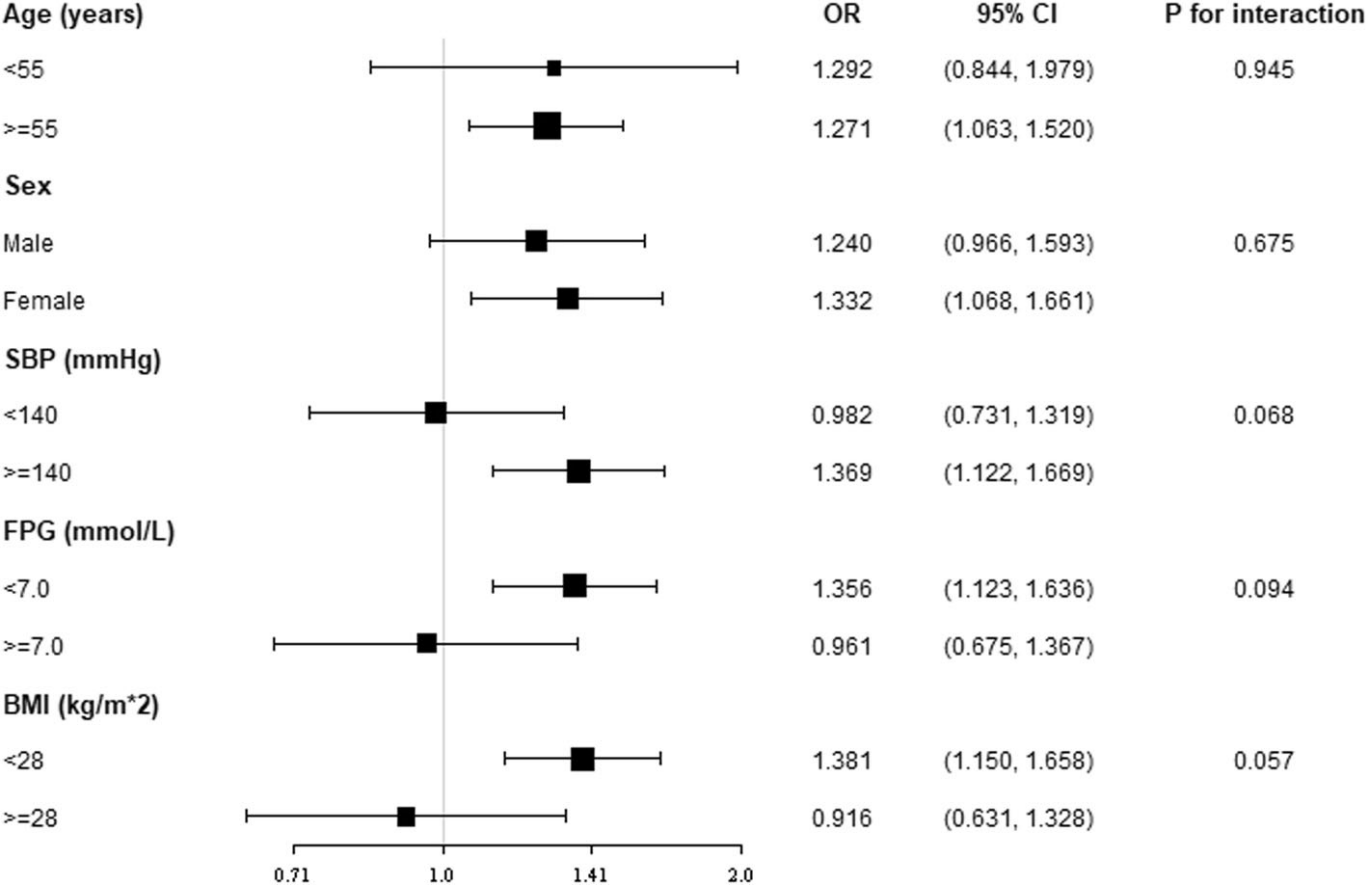
Subgroup analyses on impact of MHR on the prevalence of ischemic stroke. The dots and lines indicate the estimates of the odds ratios of ischemic stroke for each SD increment of MHR and the corresponding 95% confidence intervals, respectively. The model adjusted for age, sex, race, education level, family annual income level, physical activity, current smoking, current drinking, diet score, serum creatinine, systolic blood pressure, fasting plasma glucose, body mass index, and lipid-lowering drug, except for the variable that is stratified

To confirm the findings in logistic models were robust to potential confounders, we conducted stratified analysis by subgroups defined by covariates that had been demonstrated to have major roles in affecting stroke risk, including age, sex, SBP, FPG, and BMI (Fig. 3). All of these analyses were adjusted for age, sex, race, education, family income and physical activity level, current smoking and drinking status, diet score, Scr, SBP, FPG, BMI, and lipid-lowering drug except for the covariate that was stratified. Figure 3 revealed a highly consistent pattern: the risk for ischemic stroke increased with greater MHR regardless of subgroups (all P for interaction> 0.05).

The areas under the receiver operator characteristic curves of the multivariable logistic regression models are shown in Table 3. For ischemic stroke, the baseline logistic regression model with clinical risk factors (age, sex, current smoking, current drinking, Scr, SBP, FPG and BMI) had a C-statistic of 0.802 (95% CI, 0.773–0.831). Adding MHR significantly increased (*P* = 0.042) the C-statistic to 0.808 (95%CI, 0.779–0.837). The IDI but not the category free NDI was significantly improved when MHR was added to the baseline model adjusted for clinical risk factors.

**Table 3:** Comparison of the risk stratifying ability of MHR in addition to clinical risk factors

Model	C-Statistic (95% CI)	*P* value	NRI (category free)	*P* value	IDI	*P* value
Clinical risk factors^a^	0.802 (0.773, 0.831)	Reference	Reference	Reference	Reference	Reference
Clinical risk factors + MHR	0.808 (0.779, 0.837)	0.042	0.128 (−0.013, 0.269)	0.075	0.004 (0.000, 0.007)	0.0043

C-Statistic, net reclassification improvement (NRI) and integrated discrimination improvement (IDI) were compared between models

^a^The reference model included risk factors only, including age, sex, current smoking, current drinking, serum creatinine, systolic blood pressure, fasting plasma glucose, and body mass index

## Discussion

Our study examined for the first time the relationship between the MHR and prevalence of ischemic stroke in a large community-based population. The principal findings of our study are two-fold. First, elevated MHR levels were dose dependently associated with increasing odds of ischemic stroke, even after adjusting for traditional ischemic stroke risk factors and other important covariables. MHR may serve as a clinically useful and potentially modifiable inflammation-based marker for identifying patients who are at higher risk for cardiovascular and cerebrovascular outcomes. Second, the MHR has the potential to further refine predictive risk estimation as compared to the classic clinical risk factors, suggesting a method to optimize the prevention of ischemic stroke. Taken together, targeted preventive programs that reduce the burden of inflammation and lipid abnormalities as estimated by MHR, possibly will reduce the prevalent ischemic stroke at both levels of primary and secondary prevention.

Accelerated atherosclerosis in intracranial arteries is an intrinsic nature of ischemic stroke [5–7]. Artery–artery embolism, hypoperfusion, and branch atheromatous disease are the likely mechanisms for ischemic stroke in intracranial atherosclerosis [8, 9]. Furthermore, clinical studies also employed advanced imaging techniques and autopsy to evaluate the direct correlation between intracranial atherosclerosis and ischemic stroke, and they identified the high prevalence of intracranial atherosclerosis among ischemic stroke patients in different races [10–13]. More importantly, the prevalence of intracranial atherosclerosis and mortality of ischemic stroke were extremely high, especially in Asian population [6, 10, 12, 31]. Although intracranial atherosclerosis is a good marker of general illness related to chronic diseases states that may predispose to the development of ischemic stroke, the techniques to detect intracranial atherosclerosis at now are expensive, time-consuming and unavailable in resource-poor settings. Thus, a simple, stable and cost-effective index of atherosclerosis to refine the risk stratification of ischemic stroke may be needed.

Inflammation and lipid abnormalities are two interplayed hallmarks of atherosclerosis, driving the healing response to vascular injury and allowing the initiation and growth of atherosclerotic plaque [14, 15, 32]. Monocytes act as an important source of pro-inflammatory species during atherosclerosis process [16, 17, 33]. This suggests that activated monocytes interact with damaged or activated endothelium, characterized by the overexpression of proinflammatory cytokines/adhesion molecules. Then, monocytes infiltrate into vascular sub-endothelial space and mature into macrophages [14, 15]. These macrophages further engulf oxidized LDL-C and transform into foam cells. Then the foam cells release inflammatory cytokines which recruit more monocytes into the pathological foci. Therefore, inflammatory cholesterol ester-loaded plaque is formed [34]. Hence, it has been shown that high monocyte counts are important determinant of atherosclerotic diseases, especially among persons with coronary artery disease and ischemic stroke [35–37]. On the other end of the spectrum, HDL-C facilitates the reverse cholesterol transportation from peripheral vessels to liver, reducing the lipid accumulation in peripheral [38, 39]. Moreover, HDL-C neutralizes the pro-inflammatory and pro-oxidant effects of monocytes by inhibiting LDL-C oxidation in vascular wall and preventing monocyte recruitment into vascular wall [18, 19], thereby suppressing the proliferation of monocyte progenitor cells and controlling monocyte activation [40]. As expected, these steps protect endothelial cells from inflammation and oxidative stress. Based on these mechanisms, the relationship between reduced HDL-C levels and atherosclerotic diseases, such as coronary heart disease and ischemic stroke is well established in some prospective cohorts [41, 42]. Notably, monocytes are relevant to pro-inflammatory and pro-oxidant effect, but HDL-C functions as a reversal factor during atherosclerosis process. On the assumption that monocytes and HDL-C have apparent functions in the progression or inhibition of atherosclerosis, therefore, incorporation of monocytes and HDL-C into MHR create an improved index that included HDL-C that is found at low levels in the presence of atherosclerosis, as well as monocyte frequency, which is known to increase in this setting. Also, the results of the CANTOS study provided strong support for the hypothesis that inflammation is a treatable pathogenic mechanism in atherosclerosis [32].

MHR is a newly proposed index to estimate atherosclerosis by assessing inflammation and dyslipidemia [20]. Studies have identified the utility of MHR to predict multiple atherosclerotic diseases, such as intra-stent restenosis, coronary artery disease, infective endocarditis and metabolic syndrome, with the emphasis placed on MHR as a novel risk marker of rapidly assessing systemic inflammatory response and possible endothelial dysfunction [23–25, 43]. It is probable that an increased MHR was suggestive for inflammation that exerts its primary actions in atherosclerosis development and progression and, hence, ischemic stroke. In fact, a growing body of evidence indicates the role of inflammation in the pathogenesis of ischemic stroke [44–46]. Experimentally and clinically, acute and prolonged inflammatory process have been observed in cerebral ischemic injury, that is, the rapid activation of resident cells (mainly microglia), production of proinflammatory mediators, and infiltration of various types of inflammatory cells (including neutrophils, T cells, monocyte/macrophages, and other cells) into the ischemic brain tissue [47]. During the acute phase of ischemic stroke, injured tissue releases proinflammatory mediators (cytokines and chemokines) and reactive oxygen species, eventually promoting the adhesion and migration of circulating leukocytes [48, 49]. In the subacute phase, leukocytes infiltration potentiates the local inflammatory response, characterized by increased production of cytokines and chemokines, and activation of matrix metalloproteinase, which in turn result in junctional proteins cleavage and disruption of blood brain barrier integrity, thus feeding the deleterious inflammatory loop [48, 50]. On the basis of these findings and pathophysiological role of inflammation in ischemic stroke, we hypothesized that higher MHR would be associated with an increased odd of ischemic stroke and can be utilized as an ideal marker to improve the risk stratification of ischemic stroke. Furthermore, it is known that the large artery atherosclerosis and cardioembolic causes have been acknowledged as major determinants for ischemic stroke. As mentioned earlier, high MHR levels have been observed in patients with vascular risk factors associated with atherosclerosis and cardiovascular disease [21–25, 51]. Thus, MHR has a documented role in these processes or may modify the risk of ischemic stroke by modulating the conventional risk factor pathway. Importantly, atrial fibrillation (AF) is the most frequent reason for cardioembolic stroke. In a prospective and observational study with 402 cases of symptomatic AF, elevated MHR was correlated with an increased recurrence of after cryoballoon-based catheter ablation [21]. Taken together, the effects of MHR levels on ischemic stroke may be mediated through components of the inflammation and pathways of atrial fibrillation. Hence, it might be speculated that high MHR ratio may represent an additional independent risk factor for ischemic stroke.

Our observation of a positive association between MHR and the odds of having ischemic stroke was line with the hypothesis. These findings were independent of key sociodemographic and potentially modifiable vascular risk factors in a rural community–based sample. Higher MHR levels were associated with significantly greater odds of ischemic stroke in multivariable adjusted models. In a recent prospective study among 397 individuals with current smoking and 515 age-matched healthy participants without history of smoking, higher MHR levels have been found to be associated with cigarette smoking [51]. It is particularly notable that the strong association between elevated MHR and prevalent ischemic stroke was independent of current smoking status in our data. Additional studies are warranted to confirm the influence of amount of daily smoking and duration of smoking (pack.year) on this association. Furthermore, the smooth curve fitting validated this association was linear in the whole range of MHR value. Therefore, when using MHR as an indicator of the risk of ischemic stroke, a higher MHR value means a higher risk of ischemic stroke, and there is no threshold or saturation effect existing in this association. This discovery affirmed the stability of MHR to stratify the risk of ischemic stroke. Additionally, the association persisted in the stratification analysis of sex, age, SBP, FPG and BMI, suggesting the risk stratifying ability of MHR is applicable to a wide range of subjects.

In the ROC analysis, we observed a significant improvement for the stroke identifying ability after adding MHR into a model of clinical risk factors (including sex, age, current smoking and drinking status, Scr, SBP, FPG, and BMI). This finding evidenced the usefulness of MHR to improve risk stratification of ischemic stroke. For more accurate determination of the risk stratifying ability of MHR, we performed reclassification analysis by calculating category free NRI and IDI. The result was intriguing. Although IDI confirmed a significant risk reclassification by introducing MHR, the category free NRI remained statistically insignificant. The reason of this discrepancy can be explained by the low statistical efficiency of category free NRI [52, 53]. Algorism of category free NRI can only recognize 2 types of change in risk reclassification: upward or downward. The algorism cannot utilize the degree of the change, therefore a large upward or downward change in risk classification will cause the same category free NRI result as a small upward or downward change respectively [53], thus the category free NRI may not recognize the improvement from MHR. However, with a full usage of reclassification data, IDI successfully found out this advancement [52]. Overall, we could conclude that MHR brought a significant improvement to the risk stratification of ischemic stroke.

There are possible mechanistic explanations for the relation between MHR and ischemic stroke. First, reduced HDL-C level results in impaired reverse transportation of cholesterol from peripheral vessels to liver, leading to accumulation of lipids in arteries [54, 55]. The accumulated lipids undergo peroxidation and form oxidized phospholipids, then the products experience further hydrolyzation and derive into lysophosphatidylcholine (LPC), which mediates the major atherogenic activity of oxidized lipids [56]. As a result, elevated LPC level promotes endothelial cells to express adhesion molecules and chemo-attractants [57, 58]. Furthermore, the dysregulated lipid profiles also promote the production of circulating monocytes from hematopoietic stem cells [59, 60]. In addition, HDL-C reduction leads to decreased inhibition of the pro-inflammatory and pro-oxidant function of monocytes [18, 19]. Consequently, the over-loaded pro-inflammatory monocytes are recruited to the pathologic foci by endothelial cells released substances and then maturate into macrophages [61, 62]. These macrophages convert into foam cells through internalization of accumulated lipids. The foam cells further transform into a key component of atherosclerotic core through necrosis [15, 63]. Finally, the progressively aggravated intracranial atherosclerosis plays a critical role in the formation of artery to artery embolism, hemodynamic hypoperfusion, local branch occlusion and in-situ thrombotic occlusion, which are fundamental mechanism of ischemic stroke [64].

There are several study limitations. Due to the cross-sectional nature of our study, and no causal conclusions can be made about the temporal relationship of MHR with prevalent ischemic stroke. Second, the study is representative of the general population in the rural area of northeast China that limits the generalizability of our findings to other ethnicities with different economic condition. Third, although multivariable adjustments were performed for potential confounding factors, a possible effect of various inflammatory biomarkers such as C-reactive protein, interleukin-6, and oxidative stress biomarkers cannot be excluded. Lastly, as in any observational epidemiologic study, residual confounding by uncollected or unknown risk factors can introduce bias into our results. Yet, results were robust to adjustments for multiple major risk factors. Strengths of our study include a large population-based design, well-documented and standardized data collection instruments, and a relatively large number of ischemic stroke events to power the overall study.

## Conclusions

In summary, we were able to show for the first time that MHR was an independent predictor of ischemic stroke among rural Chinese adults and demonstrated incremental predictive value over traditional clinical risk factors, thus providing clinical utility in risk stratification in subjects presenting with ischemic stroke. Our findings also contributed to the understanding of a potential role of systemic inflammation–related mechanisms in the formation of ischemic stroke and encourage further investigation of therapeutic strategies aimed at fostering a reduction in inflammation and lipid accumulation, as reflected by MHR levels.

## Data Availability

The datasets used and/or analyzed during the current study are available from the corresponding author on reasonable request.

## Abbreviations

AF: Atrial fibrillation
BMI: Body mass index
CI: Confidence interval
CNY: Chinese yuan, 1CNY = 0.15USD
DBP: Diastolic blood pressure
FPG: Fasting plasma glucose
HDL-C: High-density lipoprotein cholesterol
IDI: Integrated discrimination improvement
LDL-C: Low-density lipoprotein cholesterol
LPC: Lysophosphatidycholine
MHR: Monocyte count to high-density lipoprotein cholesterol ratio
NCRCHS: Northeast China Rural Cardiovascular Health Study
NRI: Net reclassification improvement
OR: Odds ratio
ROC: Receiver operating characteristic curve
SBP: Systolic blood pressure
Scr: Serum creatinine
SD: Standard deviation
WC: Waist circumference
